# Adult B lymphoblastic leukaemia/lymphoma with hypodiploidy (-9) and a novel chromosomal translocation t(7;12)(q22;p13) presenting with severe eosinophilia – case report and review of literature

**DOI:** 10.1186/1756-8722-2-26

**Published:** 2009-06-21

**Authors:** Farhat Abbas Bhatti, Iftikhar Hussain, Muhammad Zafar Ali

**Affiliations:** 1 Department of Haematology, PNS Shifa Hospital, Karachi, Pakistan

## Abstract

Patients suffering from adult acute lymphoblastic leukemia are acutely ill and present most commonly with fever, pallor, bleeding, lymphadenopathy, hepatosplenomegaly and presence of lymphoblasts in the peripheral blood and bone marrow. We describe a rare presentation of acute lymphoblastic leukemia, in a young adult male who had vague and minimal symptoms with mild splenomegaly. There was severe eosinophilia along with absence of blasts in the peripheral blood, and 40% blasts with increase in eosinophils in the bone marrow. The blasts were positive for common precursor B cell markers on flow cytometry. The patient had a unique cytogenetic abnormality t(7;12)(q22;p13),-9, not previously described in acute lymphoblastic leukemia. He was categorized as poor risk due to failure to achieve complete remission after induction with UK ALL XII chemotherapy.

## Introduction

Severe eosinophilia, defined as eosinophil count > 5000/μl, can be seen in helminthic infections, allergic disorders, lymphoproliferative disorders, chronic myeloid leukemia, and chronic eosinophilic leukaemia[1]. A history of allergic disorders, exposure to helminthic infestations, passage of worms in feces, drug intake, weight loss, fever, cough, diarrhoea and skin rash need to be complemented with proper clinical examination to delineate the likely cause of eosinophilia. Extensive investigations, which include stool examination, chest X Ray, ultrasound abdomen, CT scan, bone marrow aspiration/biopsy and cytogenetic studies, are required to know the etiology and differentiate between 'reactive' or 'clonal' eosinophilia.' Severe eosinophilia may occur several years before the onset of haematological malignancy, like in Hodgkin lymphoma[2], and may pose a diagnostic dilemma.

Precursor B acute lymphoblastic leukemia with exaggerated eosinophilia is a rare entity with less than 50 cases reported since 1973, when it was first described by Spitzer and Garson [3,4]. In most patients, the characteristic feature of ALL with eosinophilia is the absence of blasts in the peripheral blood film. This could lead to delay in the diagnosis, if bone marrow aspiration is not done and the patient is started on steroid therapy. The most common cytogenetic abnormality encountered in acute lymphoblastic leukemia with eosinophilia is t(5;14), and is characterized by overproduction of IL-3 [5]. The latter entity is now included as 'B lymphoblastic leukemia/lymphoma with t(5;14); IL3-IGH' in new WHO classification of lymphoid neoplasms published in 2008 [6].

In the following case report, diagnosis and management of a young male is discussed who suffered from precursor B acute lymphoblastic leukemia with severe eosinophilia, and a unique cytogenetic abnormality 45,XY,t(7;12)(q22;p13),-9, reported for the first time.

## Case Description

A 31 years old male presented with history of aches and pains in whole body especially marked in temporomandibular joints, lower legs and both hip joints lasting for 1 month. He was also suffering from fatigue and generalized weakness for the same duration. There was no history of fever, allergies, skin rash, cough, urinary and bowel complaints. He is employed in Navy as a marine, and is a non-smoker, non-diabetic and non-hypertensive. He had received anti-tuberculosis treatment 3 years ago for pulmonary Koch's. At the time of his present illness, he was not taking any medications. He was living in the sailors' accommodation with his colleagues, and there was no history of handling of any pets. Both his parents and his 5 siblings were healthy, and did not have history of major illness in the past.

On physical examination he was comfortable, afebrile, and did not have any bone tenderness. There was no pallor, jaundice or lymphadenopathy. Pulse was 78/minute and blood pressure was 110/75 mmHg. The heart and lungs were normal on auscultation, and there were no murmurs or added sounds. On abdominal examination liver was not palpable, while spleen was enlarged and palpable 3 cm below left costal margin. Neurological examination did not show any abnormality.

**Figure 1:**
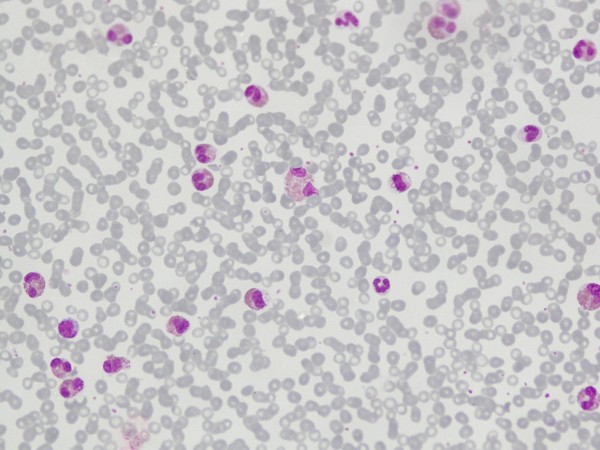
**Increased eosinophils with heterogenous features in peripheral blood film**.

His complete blood counts showed Hb: 13.6 g/dl, total leucocyte count 48 × 10^9^/l with 72% eosinophils, 21% neutrophils, 7% lymphocytes; and platelet count 167 × 10^9^/l. The absolute eosinophil count was 34.5 × 10^9^/l (34,560/cmm), and the eosinophils had heterogenous morphology in peripheral blood film (Fig 1). His ultrasound abdomen revealed splenomegaly, while there was no enlargement of para-aortic lymph nodes, or presence of abdominal/pelvic mass and abscess. 2-D echocardiography showed normal sized cardiac chambers with good left ventricular contraction. There were no vegetations on the valves, no left ventricular hypertrophy and ejection fraction was >65%. Electrocardiography revealed sinus rhythm and no evidence of any abnormality including axis deviation, ischaemia, previous infarction or heart block. Chest X-Ray showed normal lung fields and cardiac shadow. Serum bilirubin, ALT, alkaline phosphatase, urea, creatinine, sodium, potassium, uric acid and blood glucose were within normal limits. Stool routine examination did not show any ova or cysts.

**Figure 2:**
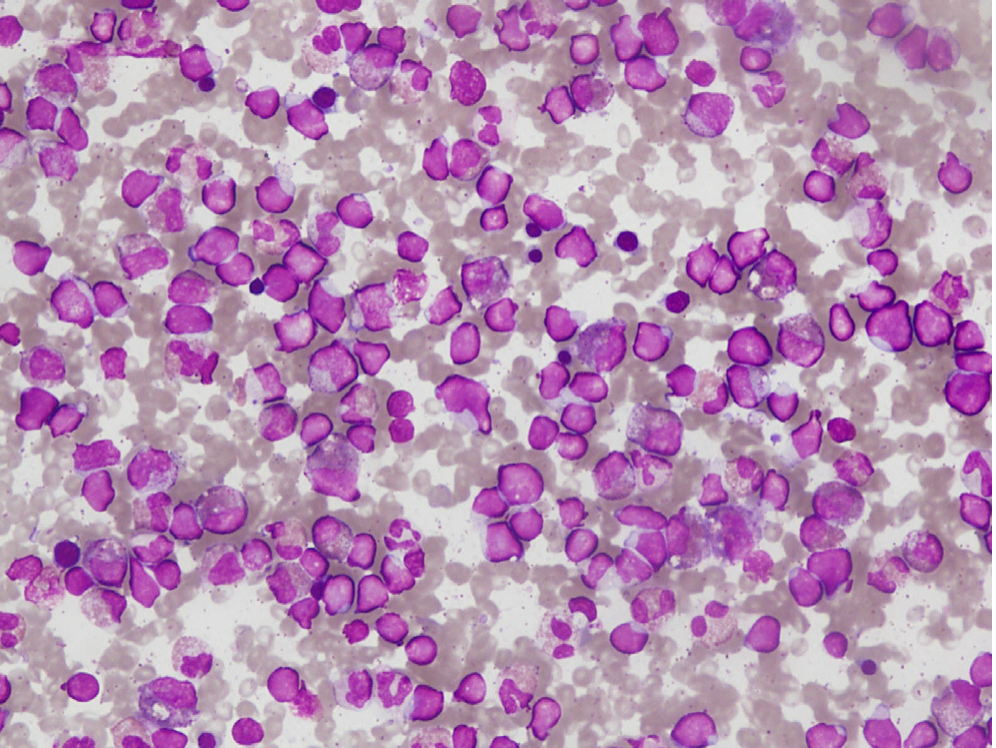
**Bone marrow aspirate of patient showing presence of blasts with high nucleocytoplasmic ratio and eosinophilic precursors**.

His bone marrow examination showed a hyperplastic marrow with depressed erythropoiesis and reduced megakaryocytes. Myelopoiesis showed increase in eosinophils and their precursors. There was infiltration by 40% blasts with high nucleo-cytoplasmic ratio, homogenous nuclear chromatin pattern and a thin rim of light basophilic cytoplasm (Fig 2). The blasts were negative for Sudan Black B, acid phosphatase but displayed occasional block positivity with Periodic Acid Schiff stain. Flow cytometric analysis (FC500 Beckman Coulter) was done after incubation of bone marrow mononuclear cells with a panel of fluorescence labeled antibodies (obtained from Becton Dickinson, USA). The blast cell population showed strong reactivity (>95%) with B-cell markers including CD10, CD19, CD20, CD22 and cCD79a.

**Figure 3:**
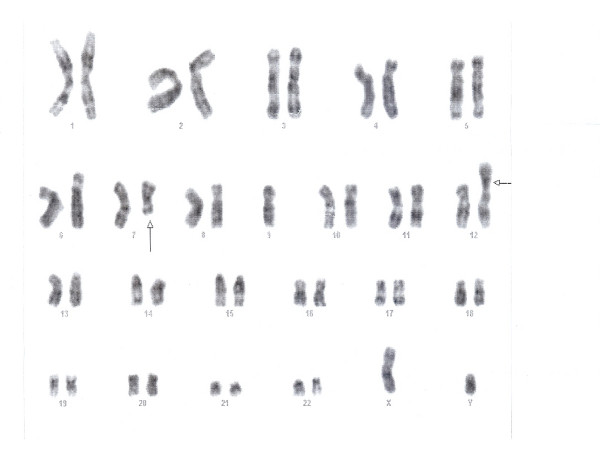
**Cytogenetics of the patient's bone marrow showing 45,XY,t(7;12)(q22;p13),-9 (20 cells were counted; 15 cells showed 45 chromosomes, while 5 cells showed 46 chromosomes)**.

The blasts did not show expression of lymphoid T markers (CD3, 5 & 7) and myeloid markers (CD 13, 33 & cMPO). HLA-DR and cTdT also showed strong positivity. There were no detectable surface immunoglobulins or cytoplasmic light chains (IgM, IgG, *kappa, lambda*). The immunophenotype was consistent with common precursor B-lymphoblastic leukaemia. Cytogenetic analysis (Fig 3) revealed karyotype 45 XY, t(7;12)(q22;p13),-9[15]/46,XY [05]. Routine examination of cerebrospinal fluid showed a protein level: 0.33 g/l, glucose: 4.4 mmol/l and occasional mature lymphocytes on Leishman's stain (There were no blasts seen in the cytospin smear).

The height and weight of the patient was 170 cm and 70 kg, respectively, and the body surface area was 1.7 m^2^. He was started on induction therapy based on UK ALL XII consisting of four drugs regimen, which included Inj vincristine 1.5 mg/m^2 ^IV on day 1, 8, 16 and 24; Inj daunorubicin 60 mg/m^2 ^IV on day 1, 8, 16 and 24; tab prednisolone 60 mg/m^2^/day × 28 days and Inj asparaginase 6000 units/m^2 ^IM days 18, 19, 20, 21, 22, 23 and 24. He was also given intrathecal methotrexate 12.5 mg on days 1 and 8. The patient tolerated chemotherapy well, except that he developed an episode of gastroenteritis on 12^th ^day of chemotherapy. He was rehydrated with IV fluids, and was administered Inj ciprofloxacin 400 mg b.d. and Inj Metronidazole 500 mg IV 8 hourly. For pneumocystis prophylaxis, he was given Co-trimoxazole 960 mg b.d 3 times a week. On the 20^th ^day of the induction therapy, the complete blood counts showed correction of eosinophilia with a total leucocyte count of 8.1 × 10^9^/l with 81% neutrophils, 12% lymphocytes, 05% eosinophils and 02% monocytes; Hb: 11.7 g/dl and platelet count: 170 × 10^9^/l. His repeat bone marrow biopsy which was done on the 28^th ^day of induction therapy showed active hematopoiesis with increase in eosinophil precursors and presence of 10% blasts in the marrow. Biochemical profile including serum urea, creatinine, electrolytes, bilirubin, ALT and uric acid were within normal limits. Serum LDH was 1254 U/ml (normal range: 250–500 u/ml). Due to his partial response to chemotherapy, re-induction therapy was given to him with the following drug combination: Inj vincristine 1.5 mg/m^2 ^day 1, Inj daunorubicin 45 mg/m^2 ^days 1 & 2; Inj etoposide 100 mg/m^2 ^days 1–5; and Inj cytosine arabinoside 100 mg/m^2 ^IV infusion 12 hourly days 1–5 and oral prednisolone 60 mg daily day 1–5 gradually to be tapered off in next two weeks. He was also given intrathecal Inj methotrexate 12.5 mg, on day 1 of second induction. The aforementioned chemotherapy regimen was repeated 4 weeks after the end of the cycle, when hematopoeitic recovery was adequate and the patient was stable. On 28^th ^day of the intensification, the patient bone marrow was done which showed normal maturation of erythroid and myeloid series with less than 5% blasts, and normal number of megakaryocytes. The patient is scheduled for evaluation at Armed Forces Bone Marrow Transplant Centre, Rawalpindi, Pakistan for stem cell transplantation from matched sibling donor.

## Discussion and Evaluation

The eosinophil count in a normal adult ranges from 0.02–0.5 × 10^9^/l (20–500/μl) [7]. Eosinophilia is classified as mild (0.5–1.5 10^9^/l), moderate (1.5–5 × 10^9^/l) and severe [8] when the eosinophil count is more than 5 × 10^9^/l. Although, the patient presented with severe eosinophilia (34.5 × 10^9^/l), there was no organ involvement, as shown by normal ECG, echocardiography, chest X-Ray, and no evidence of nervous system dysfunction or skin rash.

Different causes of eosinophilia, as shown in table 1, were excluded based upon clinical evaluation and investigations of the patient. In Pakistan, infestation with *Ascaris lumbricoides, Ancylostoma duodenale, Necator americanus *and *Echinococcus *are common causes of eosinophilia. However, there were no ova detected on stool examination and ultrasound of abdomen did not show any abnormality. This patient, diagnosed as acute lymphoblastic leukaemia, had unremarkable clinical presentation at onset, with vague symptoms and absence of fever or lymphadenopathy. His complete blood counts revealed marked eosinophilia, and interestingly absence of blasts in the peripheral blood film. The bone marrow biopsy of this patient showed presence of 40% blasts, which stained negative with Sudan Black B, and were of common precursor B-cell origin on flow cytometric analysis.

**Table 1:** Differential diagnosis of eosinophilia

Parasitic Infestations:
Ankylostoma duodenale
Necator americanus
Toxoplasma gondii
Strongyloides stercoralis
Ascaris lumbricoides
Hydatid disease

Allergic diseases
Bronchial asthma
Urticaria
Allergic rhinitis
Atopic dermatitis

Haematological malignancies
Hodgkin lymphoma
Chronic myeloid leukemia
Acute myeloid leukemia with inv (16) or t(16;16)
B lymphoblastic leukemia/lymphoma with t(5;14); IL3-IGH
Chronic eosinophilic leukemia
Myeloid & lymphoid neoplasms with eosinophilia and PDGFRA, PDGFRB and FGFR1
abnormalities

Connective tissue disorders
Rheumatoid arthritis
Serum sickness
Scleroderma

Others
Loeffler syndrome
Graft versus host disease

A variant of acute myelomonocytic leukaemia (AML-M4 Eo) [9] is associated with presence of increased number of abnormal eosinophil precursors in the bone marrow. Such patients usually have blasts in peripheral blood film and a characteristic cytogenetic abnormality of inversion of chromosome 16. Wynn et al [10] described B-cell lineage acute lymphoblastic leukaemia in a 5 year old girl, with peripheral hypereosinophilia, absence of blasts in peripheral blood film and hyperdiploid blast cell population with 5q deletion. Hypereosinophilia has also been reported in a 4 year old boy who had granular acute lymphoblastic leukaemia with a normal karyotype [11]. One case of acute lymphoblastic leukaemia with peripheral hypereosinophilia was found to have 9p21 deletion [12], on cytogenetic analysis. As discussed earlier, a common cytogenetic abnormality detected in most cases of precursor B acute lymphoblastic leukaemia with hypereosinophilia is t(5;14), which disappears during remission and reappears in relapse [13,14]. In the latter translocation, eosinophilia was secondary to overproduction of interleukin-3 by the blasts, due to activation of interleukin-3 gene on chromosome 5 after its translocation adjacent to the immunoglobulin heavy chain gene on chromosome 14 [5].

Eosinophilia may antedate the development of acute lymphoblastic leukemia by several months to 2 years, and the patients may present with urticarial lesions and other non-haematological features of hypereosnophilic syndrome (hepatosplenomegaly, cardiac lesions, CNS involvement) during this period [15,16]. Hypereosinophilia was present in our patient at the time of diagnosis of his disease, and was associated with a unique cytogenetic abnormality of t(7;12)(q22;p13),-9, present in majority of the metaphases. To the best of our knowledge, the latter translocation has not been reported earlier in adult B lymphoblastic leukemia/lymphoma. The t(7;12)(q22;p13) previously reported in infant leukemia [17], did not occur as a sole abnormality, and was accompanied by deletion of 7(q22q36). Two rare recurrent translocations {t(7;12)(q36;p13) and t(7;12)(q32;p13)} have been identified in 5/125 children, less than 18 months of age, and who were suffering from acute myeloid leukemia [18]. Involvement (deletions or translocations) of chromosome 7, especially in the region 7q22, predominantly occurs in myelodysplastic syndrome [19] and myeloid leukemias [20]. Translocation and deletion of a segment of chromosome 7 (7q22 or 7q32-q35), or loss of chromosome 7 may lead to inactivation (position effect) of tumour suppressor gene(s) or their dysfunction (at the breakpoint region), leading to transformation to myeloid leukemias [20].

The other chromosomal gene affected in this case is 12p13. More than forty translocations involving 12p13, or the ETV6 gene, and different partner chromosomes have been described in various haematopoeitic malignancies. t(5;12)(q31;p13) is a recurrent mutation which has been reported in refractory anaemia with excess blasts (RAEBt) with basophilia, acute myeloid leukemia with eosinophilia and acute eosinophilic leukemia [21]. Chromosomal abnormality affecting 12p13 and associated with severe eosinophilia, has also been described in Philadelphia negative myeloproliferative disorder, eosinophilic leukemia and acute lymphoblastic leukemia [22]. Similarly, a chimeric gene ETV6/ACS2 due to t(9;12)(q22;p13) has been reported in myelodysplastic syndrome associated with eosinophilia [23]. The ETV6 gene codes for a transcription activator, and has a span of 240 kb with eight exons [24,25]. Different segments of the ETV6 gene act as fusion partners with other genes affected in various translocations, and can result in the production of a fusion protein having a ligand-independent tyrosine kinase activity [26]. In our case, the events responsible for leukemogenesis may be related to hypodiploidy, loss of suppressor gene at 7q22, or activation of a proto-oncogene after translocation of 7q22 to 12p13 (ETV6 gene). Hypodiploidy (loss of chromosome 9) is likely responsible for poor response to chemotherapy and adverse prognosis, while eosinophilia seen in this case is most likely secondary to t(7;12). The patient was categorized in the poor risk category because of late remission (>6 weeks after induction therapy). He is thus planned to be referred for bone marrow transplantation from matched sibling with a curative intent.

## Conclusion

Marked increase of eosinophils in the blood and bone marrow can occur in precursor B-acute lymphoblastic leukemia, as a result of different cytogenetic abnormalities. In majority of the cases there is absence of blasts in the peripheral blood film. Adult B lymphoblastic leukemia/lymphoma with hypodiploidy and severe eosinophilia in blood and bone marrow, having cytogenetic abnormality t(7;12)(q22;p13),-9, is a distinct entity with poor response to chemotherapy, and a bad prognosis. Individuals with this disease should be selected for bone marrow transplantation from matched sibling/unrelated donor, once remission is achieved, for best chances of disease free survival.

## Abbreviations

ALL: Acute lymphoblastic leukemia

## Competing interests

The authors declare that they have no competing interests.

## Authors' contributions

All authors were involved in management of the patient and preparation of this manuscript. All authors read and approved the final manuscript.

## Consent

The patient has provided informed consent for the publication of this case report and accompanying images. A copy of the written consent is available for review by the Editor-in-Chief of this journal.

## References

[B1] Pardanani A, Brockman SR, Patemoster SF, Flynn HC, Ketterling RP, Lasho TL, Ho CL, Li CY, Dewald GW, Tefferi A (2004). *FIP1L1-PDGFRA *fusion: prevalence of clinicopathologic correlates in 89 consecutive patients with moderate to severe eosinophilia. Blood.

[B2] Ayyub M, Anwar M, Luqman M, Ali W, Bashir M (2003). A case of hypereosinophilic syndrome developing Hodgkin's disease after 4 years. Br J Haematol.

[B3] Spitzer G, Garson OM (1973). Lymphoblastic leukemia with marked eosinophilia: a report of two cases. Blood.

[B4] D'Angelo G, Hotz AM, Todeschin P (2008). Acute lymphoblastic leukemia with hypereosinophilia and 9p21 deletion: Case report and review of literature. Lab Hematol.

[B5] Meeker TC, Hardy D, William C, Hogan T, Abrams J (1990). Activation of interleukin-3 gene by chromosome translocation in acute lymphocytic leukemia with eosinophilia. Blood.

[B6] Swerdlow SH, Campo E, Harris NL, Jaffe ES (2008). WHO classification of tumours of haematopoietic and lymphoid tissues. (4^th ^edition).

[B7] Lewis M, Lewis SM, Bain BJ, Bates I (2006). Reference ranges and normal values. Dacie and Lewis Practical Haematology.

[B8] Rothenberg ME (1998). Eosinophilia. N Eng J Med.

[B9] Haferlach T, Winkemann M, Loffler H, Schoch R, Gassmann W, Fonatsch C, Schoch C, Poetsch M, Weber-Matthiesen K, Schlegelberger B (1996). The abnormal eosinophils are part of the leukaemic cell population in acute myelomonocytic leukemia with abnormal eosinophils (AML M4Eo) and carry pericentric inversion 16: a combination of May Grunwald Geimsa staining and fluorescence in situ hybridization. Blood.

[B10] Wynn TT, Heerema NA, Hammond S, Ranalli M, Kahwash SB (2003). Acute lymphoblastic leukaemia with hypereosinophilia: report of a case with 5q deletion and review of literature. Pediatr Dev Pathol.

[B11] Jain P, Kumar R, Gujral S, Kumar S, Singh A, Jain Y, Dubey S, Anand M, Arya LS (2000). Granular acute lymphoblastic leukaemia with hypereosinophilic syndrome. Ann Hematol.

[B12] D'Angelo G, Hotz AM, Todeschin P (2008). Acute lymphoblastic leukaemia with hypereosinophilia and 9p21 deletion: Case report and review of literature. Laboratory Hematology.

[B13] Hogan TF, Koss W, Murgo AJ, Amato RS, Fontana JA, VanScoy FL (1987). Acute lymphoblastic leukemia with chromosomal 5;14 translocation and hypereosinophilia: case report and literature review. J Clin Oncol.

[B14] Baumgarten E, Wegner RD, Fengler R, Ludwig WD, Schulte-Overberg U, Domeyer C, Schüürmann J, Henze G (1989). Calla-positive acute leukaemia with t(5q;14q) translocation and hypereosinophilia – a unique entity?. Acta haematologica.

[B15] Hill A, Metry D (2003). Urticarial lesions in a child with acute lymphoblastic leukaemia and eosinophilia. Pediatr Dermatol.

[B16] Blatt J, Proujansky R, Horn M, Phebus C, Longworth D, Penchansky L (1992). Idiopathic hypereosinophilic syndrome terminating in acute lymphoblastic leukemia. Pediatr Hematol Oncol.

[B17] Tosi S, Hughes J, Scherer SW, Nakabayashi K, Harbott J, Haas OA, Cazzaniga G, Biondi A, Kempski H, Kearney L (2003). Heterogeneity of the 7q36 breakpoints in the t(7;12) involving ETV6 in infant leukemia. Genes Chromosomes Cancer.

[B18] Slater RM, Drunen EV, Kroes WG (2001). t(7;12)q36;p13) and t(7;12)(q32;p13)-translocations involving ETV6in children 18 months of or younger with myeloid disorders. Leukemia.

[B19] Johnson EJ, Scherer SW, Osborne L, Tsui LC, Oscier D, Mould S, Cotter FE (1996). Molecular definition of a narrow interval at 7q22.1 associated with myelodysplasia. Blood.

[B20] Fischer K, Frohling S, Scherer S, Brown JM, Scholl C, Stilgenbauer S, Tsui LC, Lichter P, Döhner H (1997). Molecular cytogenetic delineation of deletions and translocations involving chromosome band 7q22 in myeloid leukemias. Blood.

[B21] Odero MD (2007). t(5;12)(q31;p13) in MDS, AML and AEL. Atlas Genet Cytogenet Oncol Haematol.

[B22] Keene P, Mendelow B, Pinto MR, Bezwoda W (2008). Abnormalities of chromosome 12p 13 and malignant proliferation of eosinophils: a nonrandom association. Br J Haematol.

[B23] Kuno Y, Abe A, Emi N, Iida M, Yokozawa T, Towatari M, Tanimoto M, Saito H (2001). Constitutive kinase activation of the TEL-SYK fusion gene in myelodysplstic syndrome with t(9;12)(q22;p12). Blood.

[B24] Wlodarska I, Mecucci C, Baens M, Marynen P, Berghe H van den (1996). ETV6 gene rearrangementsin haemopoietic malignant disorders. Leuk Lymphoma.

[B25] Gao NA, Li ZH, Ding BT, Chen Y, Wang YS, Qiao Y, Guo NJ (2008). Expression of ETV6 rearrangement in a subject with acute myeloid leukemia-M4Eo. Chin Med J.

[B26] Wai DH, Knezevich SR, Lucas T, Jansen B, Kay RJ, Sorensen PH (2000). The *ETV6-NTRK3 *gene fusion encodes a chimeric protein tyrosine kinase that transforms NIH3T3 cells. Oncogene.

